# Neonatal- maternal separation primes zymogenic cells in the rat gastric mucosa through glucocorticoid receptor activity

**DOI:** 10.1038/s41598-018-28223-1

**Published:** 2018-06-29

**Authors:** Daniela Ogias, Isadora C. Rattes, Larissa Y. M. Hosoya, Juliana G. Zulian, Chao Yun Irene Yan, Patrícia Gama

**Affiliations:** Department of Cell and Developmental Biology, Institute of Biomedical Sciences, University of Sao Paulo, São Paulo, Brazil

## Abstract

Neonatal- Maternal Separation (NMS) deprives mammals from breastfeeding and maternal care, influencing growth during suckling- weaning transition. In the gastric mucosa, Mist1 (encoded by *Bhlha15* gene) and moesin organize the secretory apparatus for pepsinogen C in zymogenic cells. Our current hypothesis was that NMS would change corticosterone activity through receptors (GR), which would modify molecules involved in zymogenic cell differentiation in rats. We found that NMS increased corticosterone levels from 18 days onwards, as GR decreased in the gastric mucosa. However, as nuclear GR was detected, we investigated receptor binding to responsive elements (GRE) and observed an augment in NMS groups. Next, we demonstrated that NMS increased zymogenic population (18 and and 30 days), and targeted Mist1 and moesin. Finally, we searched for evolutionarily conserved sequences that contained GRE in genes involved in pepsinogen C secretion, and found that the genomic regions of *Bhlha15* and *PgC* contained sites highly likely to be responsive to glucocorticoids. We suggest that NMS triggers GR- GRE to enhance the expression and to prime genes that organize cellular architecture in zymogenic population for PgC function. As pepsinogen C- pepsin is essential for digestion, disturbance of parenting through NMS might alter functions of gastric mucosa in a permanent manner.

## Introduction

Parenting represents the connection established between parents and their offspring through nurturing, feeding, protection, genetics, microbiome and epigenetics^1–3^. In mammals, mother’s milk is the essential part of this link, and among humans, many times, breastfeeding can be neglected due to social and economical conditions^4^. Neonatal-maternal **s**eparation (NMS) represents a situation in which the offspring is chronically deprived of maternal care, both in terms of breastfeeding and behavior. Because such condition is common in many countries^4^ NMS is also used experimentally as a model to study effects on development of pups^5^ and babies^6^, and the putative consequences to adult life^7^. Different reports demonstrate that NMS changes hypothalamus- pituitary- adrenal (HPA) axis and triggers anxiety and cognitive responses^8–12^. Additionally, the practice of NMS before weaning increases the permeability^13^ and alters morphology^14^ and motility in the gut^15^, especially in the colonic intestinal epithelium in adulthood, elevating the risk of intestinal diseases^16–19^.

As mentioned above, NMS effects depend on the HPA axis, and so corticosteroid responses might be directly involved. Accordingly, Ryu *et al*.^20,21^ observed that the fasting- re- feeding cycles, that are part of NMS, increased corticosterone levels and induced higher consumption of chow in young- adult rats. After working with different models to alter feeding conditions^22–25^, we observed that short- term fasting increases corticosterone levels in pups and adult rats, but it also changes glucocorticoid availability by altering the binding to its carrier globulin (CBG), in a way that pups are protected from high corticosterone concentration through elevated CBG binding^26^. Such response seems to be specific of fasting condition, as it was not detected in early- weaned rats^25^.

Corticosterone functions depend on different elements that include the availability and activity through glucocorticoid receptor (GR)^27–31^. GR was first described by Munck and Brinck- Johnsen^32^ and it is a cytosolic receptor that once activated, rapidly enters the nucleus (t_1/2_ = 5–10 min)^33^ to bind specific sequences in target genes, which are known as glucocorticoid responsive elements (GRE)^31,34^. In the gastric mucosa, GR are expressed during postnatal development and are responsive to circulating glucocorticoids^25,35^. In mice and rats, the morphological and functional maturation of gastric mucosa occur concomitant with postnatal development and the glands reach a final configuration by the end of the third week^24,36,37^. The whole growth process involves the action and interaction of milk- born molecules, extension of suckling phase, corticosterone activity, genetic program and microbiota. In each epithelial cell, a set of genes prepares the many steps necessary for differentiation, so that in parietal cells, *Atp4b* encodes the proton pump (H^+^-K^+^/ATPase), which transports ions, acidifying the lumen; in mucous neck cells (MNC), *Muc6* encodes the mucin secreted to protect the epithelium, and as MNC turn to zymogenic cells (ZC), the transition is characterized by expression of genes that arrange the cell apex and secretory apparatus^35,38–40^. In ZC, the *moesin* gene encodes a cytoskeleton linker protein that is segregated to the apical membrane of gastric cells where it helps to maintain cell polarity^41^, and *Bhlha15* encodes Mist1 transcriptional factor that is involved in organization of pepsinogen granules for secretion^39^. Previously, we showed that in rats, early weaning accelerates the differentiation of MNC^24^ and ZC^35^. Of note, we demonstrated that corticosterone is an important element in the machinery that coordinates cellular morphological maturation, and we suggested that the changes are maintained to adulthood, indicating a reprogram of growth^35^.

As gastric maturation occurs during postnatal development and in parallel to suckling to weaning transition, our hypothesis was that NMS might affect the cells and the action could be triggered by corticosterone activity. Currently, our aim was to evaluate the impacts of NMS on body weight gain, total corticosterone plasma levels, GR expression, and mainly, receptor translocation and function in the nucleus of gastric gland epithelial cells. As responses to corticosterone during development can induce sustained effects through adult life^35^, we also compared the effects in young- adult rats to check whether NMS might be imprinting the gastric mucosa.

We found that as corticosterone levels increased from 18 days onwards in NMS animals, GR expression and distribution decreased in the gastric epithelium. However, nuclear binding to GRE augmented in NMS pups and young adults, as well as regulatory genes and proteins (Mist 1 and moesin) involved in pepsinogen C secretion in zymogenic cells. Additionally, we observed that genomic regions of *Bhlha15* and *PgC* contained sites that might be highly responsive to glucocorticoids. Therefore, our study was the first to show that although GR expression and protein levels were down- regulated by NMS, receptor activity measured through GRE- binding assays increased as well as expression of genes that encode the proteins essential for digestion. Moreover, we suggested that the genes involved in the coordination of zymogenic cell differentiation and pepsinogen C synthesis might be reprogrammed by NMS through corticosterone activity. The clinical consequences of the changes shown should be explored in order to guide future directions in different fields.

## Results

### NMS decreases body mass during postnatal development

**Figure 1:**
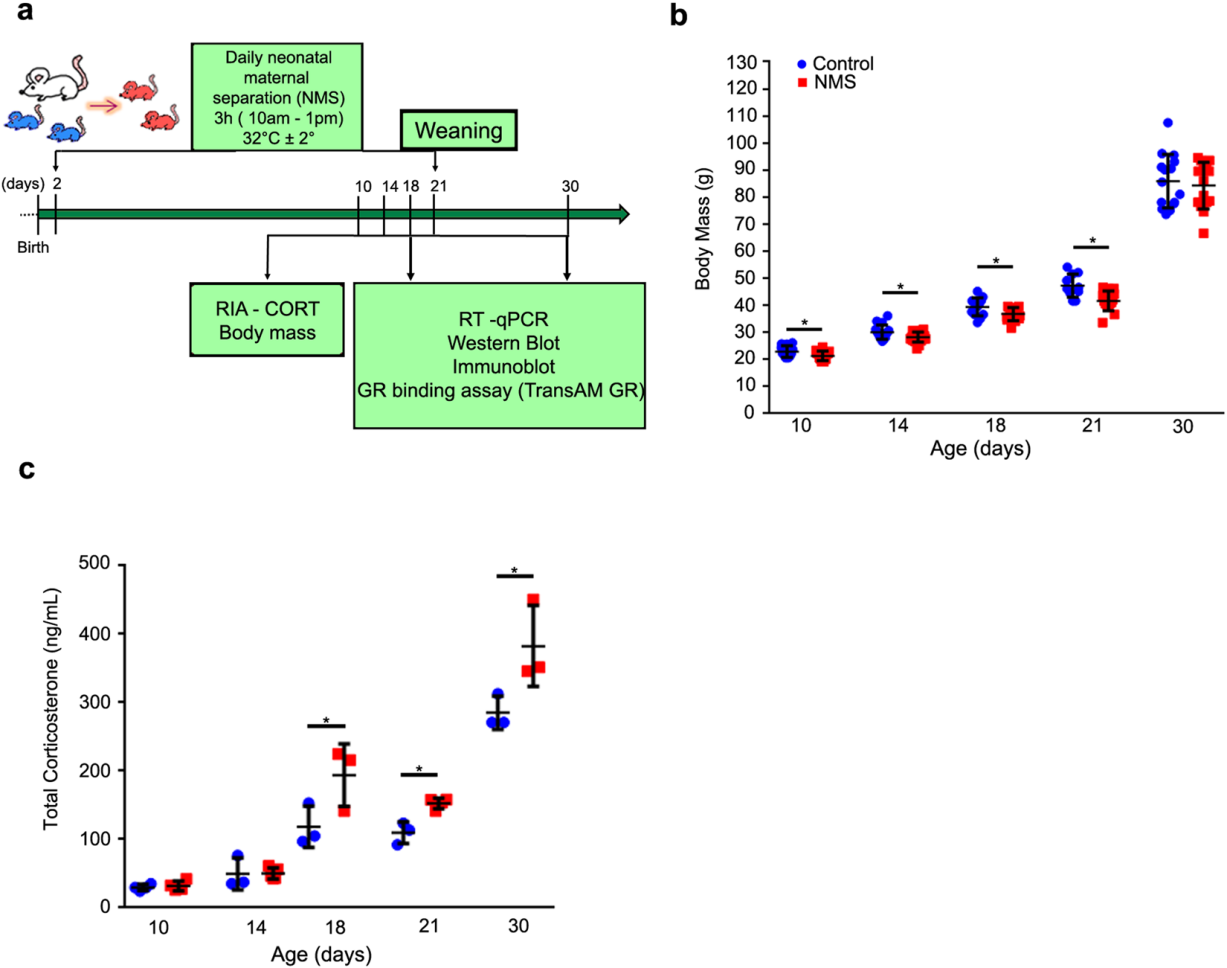
Neonatal maternal separation effects on body mass and total corticosterone plasma levels. (**a**) Representative scheme of experimental design. (**b**) Body mass (g) was registered at sample collection. (**c**) Total corticosterone plasma level was measured by RIA (ng/mL). Values were individually represented, and lines indicate means ± SD for control (blue) and NMS (red) groups. *P < 0.05 after Student *t* test. (n = 15/group/age for body mass and n = 4/group/age for total corticosterone levels).

As neonatal maternal separation is characterized by daily deprivations of feeding and maternal care (3 h/day until weaning), it might lead to changes in body mass gain, and so, we tracked it in suckling (10, 14 and 18 days), weanling (21 days) and young- adult rats (30 days) (Fig. 1a). We observed that body mass was reduced in NMS pups (10 to 21 days) when compared to control counterparts that suckled normally (Fig. 1b). After cessation of treatment at weaning (21 days), rats gained weight, and body mass was similar between groups in young- adults (30 days) (Fig. 1b).

### NMS increases corticosterone levels and decreases GR in the gastric mucosa in pups and young- adults

Corticosterone is essential for cellular and metabolic functions, and its levels are regulated by different mechanisms^27–31,42,43^. In rodents, the period from postnatal day 2 to 14 is considered hypo- responsive to stressful conditions, and so, the activity from hypothalamus- pituitary- adrenal (HPA) axis is reduced^44–46^. Because our hypothesis was based on the effects of NMS on corticosterone activity on gastric epithelial cells, we first measured its concentration in the plasma. We found that 10- and 14-day- old pups did not show changes in hormone levels (Fig. 1c), but interestingly, after that, at 18, 21 and 30 days, the levels of plasma corticosterone increased when NMS and control rats were compared (Fig. 1c).

**Figure 2:**
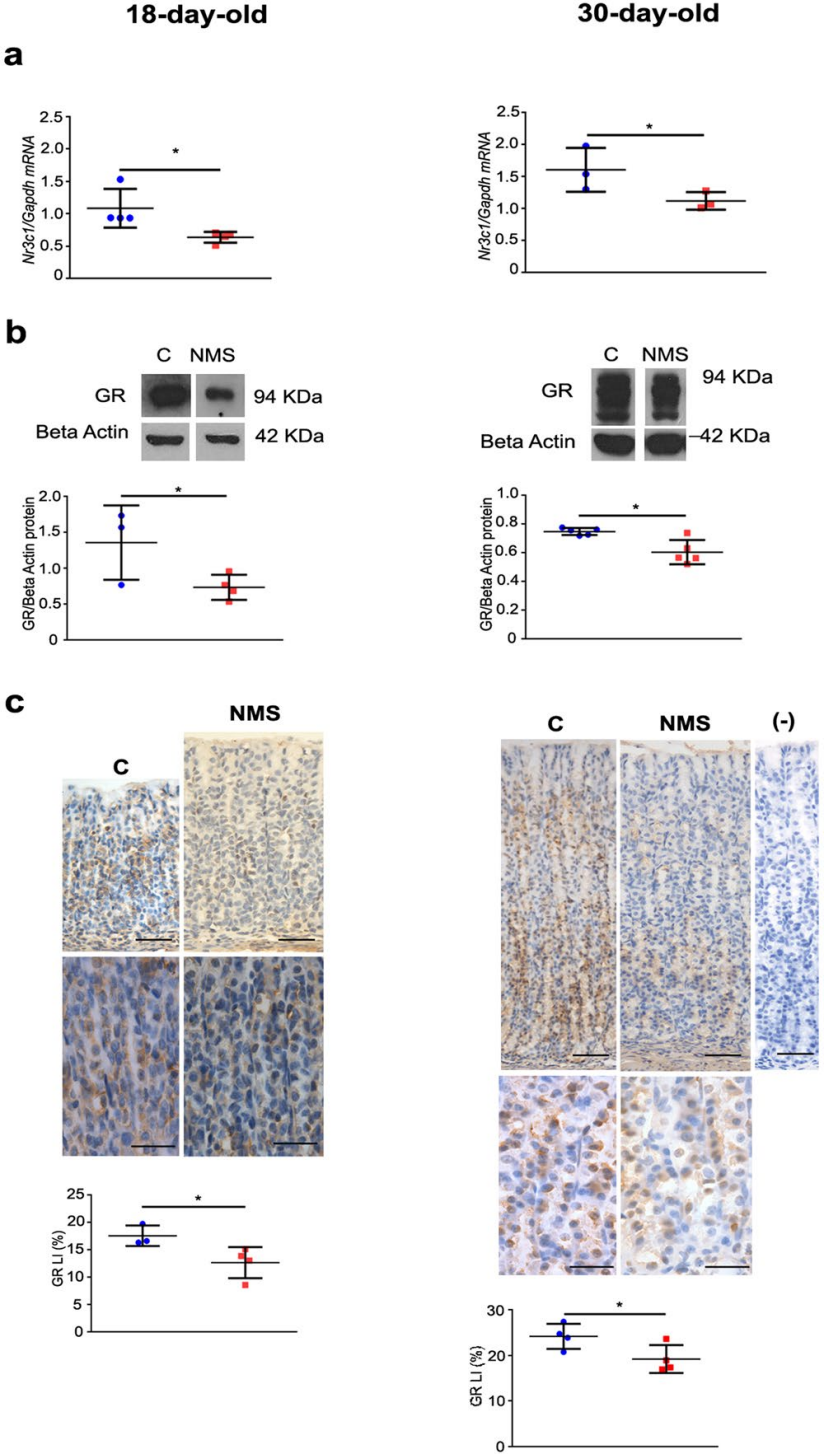
NMS effects on GR at gene expression (Nr3c1), protein levels and distribution in the gastric mucosa at 18 and 30 days. (**a**) Gene expression was evaluated by RT- qPCR and shown as fold change of *Gapdh*. (**b**) GR bands were cropped after immunoblot (Supplementary Fig. 4) to represent control and NMS groups. Protein levels were determined after comparison to the loading control with Beta- actin. (**c**) Representative photomicrographs of GR immunohistochemistry used to analyze receptor distribution. Labeling index (LI) (%) was obtained after cell counting. Negative control is devoid of any immunostaining. Bars = 50 µm; high magnification at 30 days: bars = 25 µm. Values are individually represented as well as means ± SD for control (blue) and NMS (red) groups. *P < 0.05 as compared to control group by Student *t* test.

Next, we evaluated the effects of NMS on GR in the gastric mucosa at 18 and 30 days. During third postnatal week, the receptor is active in gastric epithelial cells^23,25,35^ but levels and function might not be exclusively regulated by corticosterone. Therefore, we studied both distribution and function. At first, we observed that NMS decreased GR at gene expression (*Nr3c1*) and protein levels (Fig. 2a,b). Importantly, though treatment was stopped by weaning (21 days), the reduction was still detected at 30 days (Fig. 2a,b).

We also analyzed the tissue distribution of GR in terms of gland regions, and we found that immunolabeled cells are spread along the gastric gland at 18 days whereas at 30 days, they are more restricted to the neck- base transition (Fig. 2c). NMS did not alter this distribution. Cytoplasmic and nuclear immunostaining were observed, but not quantified. Upon determination of the GR labeling index, we verified that NMS decreased the distribution of GR-positive cells at 18 and 30 days. So, both in pups and young- adult rats, we demonstrated that NMS increased plasma corticosterone and decreased GR in the gastric mucosa.

### Increased GR nuclear binding in the gastric mucosa after NMS

As demonstrated before^23,25,47^ and confirmed above, GR is expressed in the rat gastric mucosa during postnatal development. In the cell, inactive GR is a cytoplasmic protein that is part of a multi- protein complex^33^. Once activated by glucocorticoids, GR rapidly translocates to the nucleus and can regulate gene expression through at least three different pathways: (*a*) directly binding to GRE^31,48,49^; (*b*) tethering to other DNA- bound proteins that act as transcriptional factors, and (*c*) in a composite manner, when it binds to GRE and associates to other transcriptional elements^28^.

**Figure 3:**
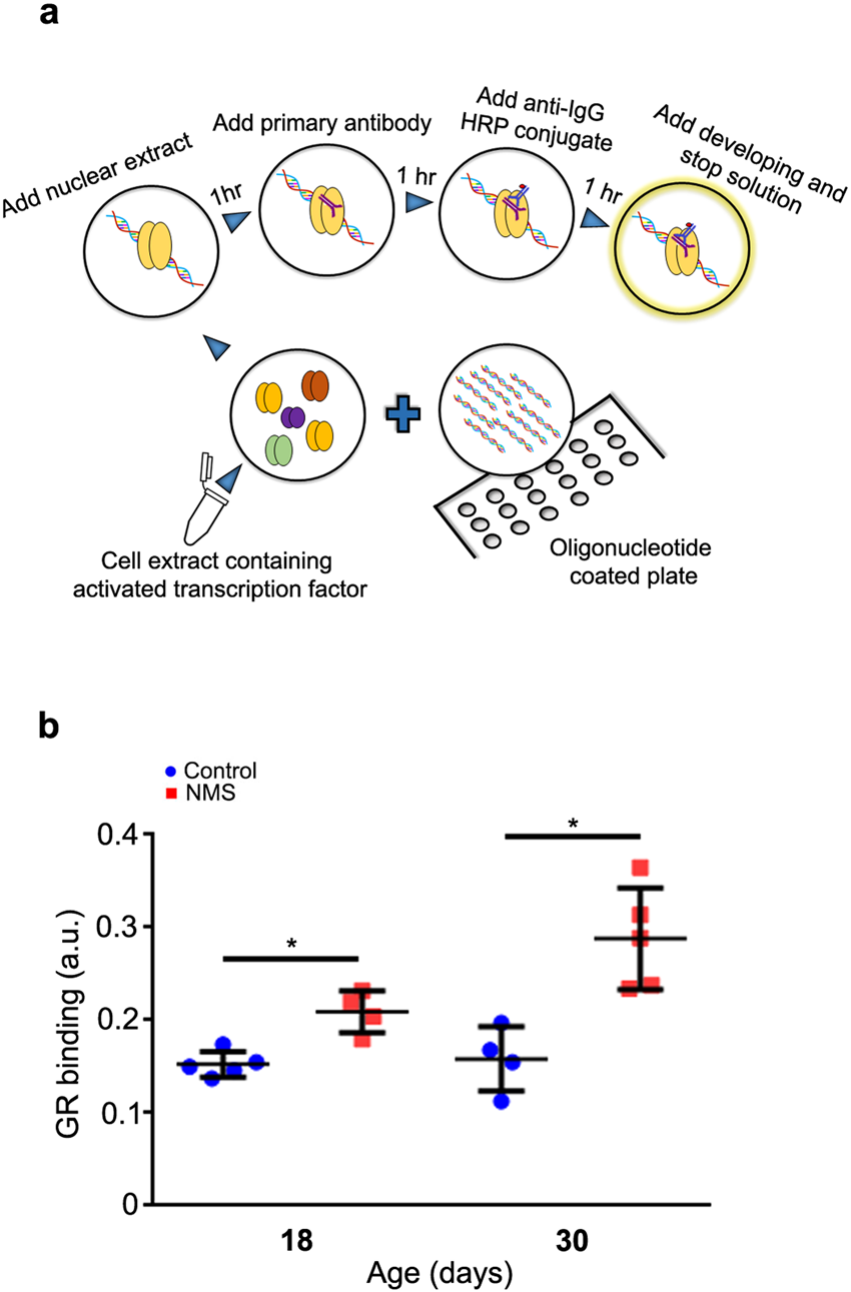
NMS effects on nuclear GR binding in the gastric mucosa at 18 and 30 days. (**a**) Flow chart of the TransAM^TM^ GR binding assay. Activated transcription factor in nuclear extract binds to the consensus- binding site on the oligonucleotides immobilized in the well, and it is detected after incubation with anti- GR, followed by secondary HRP – conjugated antibody and reaction development. (**b**) GR nuclear binding (a.u.) for control (blue) and NMS (red) groups. Values are individually represented as well as means ± SD. *P < 0.05 when compared to control group by Student *t* test.

Different disorders affect glucocorticoid- GR- GRE cascade^49^ and lead to changes in development and function^50^. As we detected high corticosterone concentration and reduced levels of gastric GR after NMS, we evaluated the effects of NMS on GR binding to GRE in the gastric mucosa at 18 and 30 days. By using a binding assay (Fig. 3a), we observed that NMS increased the response at both ages (Fig. 3b), and comparatively, the effect was more prominent at 30 days.

### NMS targets zymogenic cells

**Figure 4:**
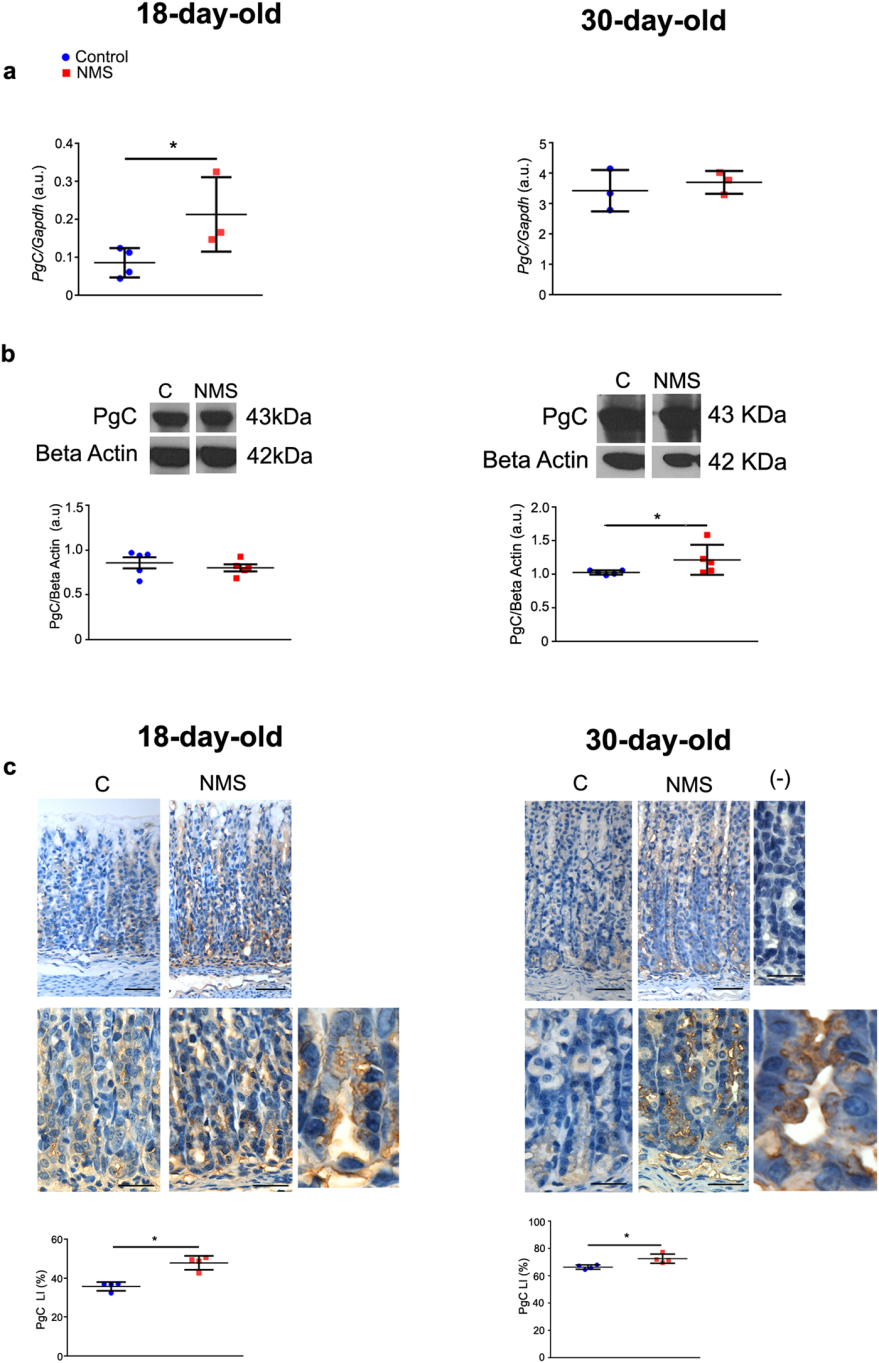
NMS effects on pepsinogen C at gene expression (*PgC*), protein levels and distribution of zymogenic cells in the gastric mucosa at 18 and 30 days. (**a**) Gene expression was evaluated by RT- qPCR and shown as fold change of *Gapdh*. (**b**) Detection of pepsinogen C and pepsin protein levels after immunoblot. Bands were cropped to represent control and NMS groups (whole blot in Supplementary Fig. 4). Densitometry was normalized to Beta-actin loading control. (**c**) Representative photomicrographs of the base region of gastric glands, showing PgC immunohistochemistry. Negative control is devoid of any reaction. Bars = 50 µm; high magnification at 30 days: bars = 25 µm. For both ages, the last panel shows X2 digital zoom to depict cellular details. Labeling index (LI) (%) was obtained as described for control (blue) and NMS (red) groups at 18 and 30 days. Values are individually represented as well as means ± SD. *P < 0.05 as compared to control group by Student *t* test.

The results above showed that NMS increased plasma corticosterone, and reduced GR distribution in the gastric mucosa, but effectively, receptor’s activity augmented, as binding to GRE increased in pups and young- adult rats. Recently, we demonstrated that during early weaning, glucocorticoids take part of differentiation of zymogenic cell population^35^, which derives from mucous neck cells^38,40^. Because NMS is also a condition that disturbs feeding and behavior during development and the gastric mucosa is responsive to endogenous corticosterone^23,25,35^ currently, we studied the molecules involved in the characterization of zymogenic cells. Also, to compare to response of this population to others components in the gastric mucosa, we evaluated the expression of genes involved in parietal and mucous neck cell activity (Supplementary Fig. 1), and *Sgk1* that is a target of GR in gastric mucosa. We observed that *Muc6* was increased both at 18 and 30 days, whereas *Atp4b* was not changed. *Sgk1* increased in pups, but not in young adults (Supplementary Fig. 1). In zymogenic cells, we found that NMS increased the expression of pepsinogen C gene (*PgC*) at 18 days, but the effect was not maintained at 30 days (Fig. 4a). The expression of gastric intrinsic factor (*Gif*) (another product of ZC) was not altered in these cells (Supplementary Fig. 1).

After protein synthesis, PgC is stored in granules and secreted to the lumen, where it is activated to pepsin. We observed that levels augmented only at 30 days (Fig. 4b). Because differences between gene expression and protein concentration could be related to product localization, whether cytoplasmic or luminal, we used immunohistochemistry to identify the cells and we estimated the labeling index. We found that at 18 and 30 days, NMS increased the distribution of zymogenic cells that were synthesizing PgC, as detected by granular cytoplasmic immunostaining (Fig. 4c).

**Figure 5:**
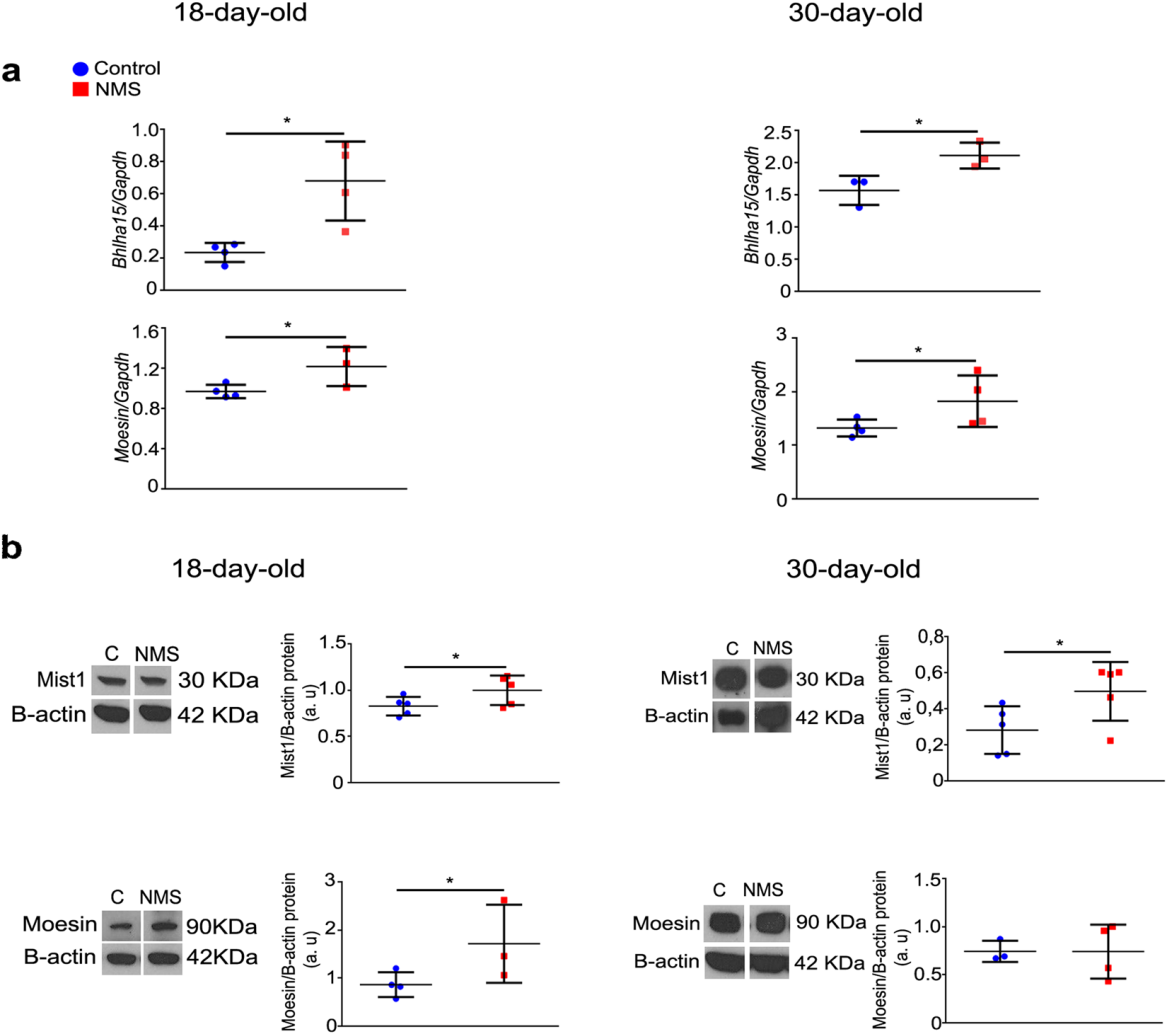
NMS effects on Mist1 and moesin at gene expression and protein levels in the gastric mucosa at 18 and 30 days. (**a**) Expression of *Bhlha15* and *moesin* genes as evaluated by RT-qPCR and shown as fold change of *Gapdh*. (**b**) After immunoblot, bands for Mist1 and moesin were cropped to represent control and NMS groups (whole blot in Supplementary Fig. 4). Protein levels were determined after comparison to the loading control with Beta- actin. Values are individually represented as well as means ± SD for control (blue) and NMS (red) groups. *P < 0.05 as compared to control groups by Student *t* test.

Zymogenic cells derive from mucous neck cells and the differentiation process involves Mist1 transcriptional factor^38,51^ and moesin protein, which is important in the organization of secretion apparatus, from mucous into serous pattern^41^. In order to verify whether NMS would influence cell reshaping, we evaluated the expression levels of Mist1 and moesin, and observed that treatment increased expression (*Bhlha15* and *moesin* genes, respectively) at 18 and 30 days. The protein concentration of Mist1 was higher after NMS in pups and young- adult rats, whereas moesin increased only in pups (Fig. 5).

After detecting that NMS increased GR nuclear binding and that it affected the differentiation of gastric zymogenic cell population, we searched for evolutionarily conserved genomic regions (ECR) in *Bhlha15* and *PgC* genes that might function as GREs. We aligned human, rat and mouse sequences, identified those that were conserved and searched for GR binding sites in both coding and non- coding regions. We found several putative GREs for each gene (Supplementary Fig. S2 and Table S2).

The results presented above suggested that GR might be involved with zymogenic cell differentiation and function, and so we used sections from 30- day- old rats to compare the localization of GR immunostaining with the distribution of zymogenic and parietal cells, which identify the gland regions (Supplementary Fig. 3). Different sections and animals were studied and GR was more concentrated at neck- base transition, where cells were immunolabeled for Mist1 and few parietal cells (DBA- stained cells) were detected.

## Discussion

As neonatal- maternal separation impacts development, metabolism and behavior^10,12,52–54^, and the growth of gastric mucosa might be affected, we currently investigated some important cellular and tissue aspects that could be targeted by corticosterone activity in rat pups and young adults.

The first parameter to be checked was weight gain, and we found that up to weaning, NMS reduced body mass, when compared to counterparts that were suckling regularly, but after that period, as rats fed on chow and were not dependent on maternal care, weight gain was restored. Though we did not attempt to measure milk intake during NMS, which would be technically doubtful, we considered that as pups were weaned, the NMS group was able eat and to recover, especially because they were released from the disturbing condition induced by treatment.

Next, we evaluated corticosterone levels, and we observed that NMS did not alter the concentration at 10 and 14 days, corroborating studies that showed that such treatment does not influence hormone levels during hypo- responsive period^53^, which extends through the second postnatal week in rats^9,23,25,45,46^. Moreover, the lack of corticosterone response at 10 and 14 days also represents a protective mechanism to guarantee the ontogenesis of systems and organs, including the gastrointestinal tract. However, at 18, 21 and 30 days, we found that NMS increased corticosterone levels, suggesting that even though treatment ceased at 21 days, the effects were still detected in young adults, and the circulating glucocorticoid might interfere in cellular functions. Such high steroid concentration induced a negative feedback over GR in gastric epithelial cells, as both expression and distribution were reduced. When compared to other feeding and nutritional models, NMS differs from short- term fasting in pups, in which high corticosterone is concomitant with increased gastric GR and CBG binding, which renders a very low traffic into cellular nucleus, and so a reduced final activity^23^.

As the function of corticosterone- GR system has been shown to be important in the growth and differentiation of gastric mucosa, and results were suggestive of negative feedback during NMS, we tested whether nuclear receptors were binding to GRE, aiming to determine effectiveness. Of note, through morphology, we identified cytoplasmic and nuclear immunostaining, indicating different levels of activity in gastric cells. So, we compared GR- GRE interaction at 18 and 30 days, which represent important stages of growth in rats, and found that GR binding to GRE increased in both ages. We also registered high *Sgk1* expression in NMS group in pups, indicating high GR activity on epithelial cells. Therefore, the practice of NMS during the first weeks of development induced alterations in corticosterone response that were noted during treatment (18 days) and in young adults as well, suggesting long- lasting effects. Similarly, early weaning (another disturbance of feeding and maternal- neonatal interaction) reprograms essential elements gastric growth^24,55^ through a mechanism that involves the direct action of corticosterone on secretory cells^25,35^. We should mention that mucin secreting cells (both in the surface and neck of the gland) were investigated, and only *Muc6* expression increased after NMS. The morphological parameters evaluated for cells distribution were not affected by the change of feeding pattern (data not shown).

The conversion of pepsinogen C into pepsin is essential for digestive functions^56^ and *PgC* transcription increases in parallel to gastric ontogenesis^57^ during suckling- weaning period, in a way that by the end of third postnatal week, PgC is the main product of zymogenic cells^31,35,57,58^. Because NMS augmented GR- GRE binding in the nucleus of gastric epithelial cells and the zymogenic cell population might be a target for such response, we evaluated the expression, levels and distribution of PgC. We found that at 18 and 30 days, NMS increased PgC content, as a whole, though a differential regulation was observed on mRNA and protein expression in each age individually. As mentioned before, the reorganization of secretory apparatus from mucous neck cells (glycoproteins) into ZC during transition depends on the expression of scaling factors^38,40,41,51,59^, and among them, we studied Mist1 and moesin. We verified that NMS increased both factors at mRNA and protein levels, suggesting that *Bhlha15* and *moesin* genes are kept activated by interference in maternal interaction during suckling period. Furthermore, as both *Bhlha15* and *PgC* genomic regions contained multiple putative GRE, we considered that NMS might affect the differentiation of zymogenic cells as well as pepsinogen synthesis through corticosterone response. Also, the proximity of GR to ZC and parietal cells at the base is suggestive of an important signaling function in these cells. So, our current results are suggestive of a priming action of NMS through GR onto genes that regulate the secretory apparatus in zymogenic cells and thus, the function of PgC in the stomach.

In brief, we showed that neonatal- maternal separation affected body weight gain throughout postnatal development and increased the total corticosterone levels from 18 days onwards; although GR expression and protein levels were down- regulated, the receptor activation through binding to GREs increased; the expression of genes that encode the proteins essential for digestion was augmented by NMS, and levels remained high in young- adult rats. We concluded that the genes involved in the coordination of zymogenic cell differentiation and pepsinogen C synthesis might be reprogrammed by neonatal- maternal separation through corticosterone activity. Interestingly, these findings brought new data to studies conducted before on the important roles of corticosterone on pepsinogen levels^35,60–62^, and more importantly, we were able to combine them to the advances of gastric gland architecture described recently and mentioned above, and to the delicate scenario of parenting and maternal- neonatal interactions. The clinical consequences of the changes currently shown should be explored in order to guide future directions in different fields.

## Material and Methods

### Rats and neonatal maternal separation

Wistar rats were obtained from the Animal Colony at the Department of Cell and Developmental Biology (Institute of Biomedical Sciences -ICB, University of São Paulo). Pregnant females were kept in individual cages at 22 °C under 12 h light and 12 h dark cycle (lights on at 06h00) with free access to food and water. Delivery was set as day 0 and litters were culled to 9 pups on the 2^nd^ postnatal day. All procedures were performed according to the guidelines of National Council of Ethics with Animals (CONCEA) and the protocols were approved by the Ethical Committee for Animal Use from ICB at University of Sao Paulo (17/2013). Experiments were conducted in order to minimize the number of animals and to allow best conditions during treatment.

On the 2^nd^ postnatal day, pups were randomly assigned into two groups, in which they either remained with the dam (control, C) or were separated from her (neonatal maternal separation, NMS). The control group suckled freely until weaning (21 days), whereas NMS pups were separated from the dams 3 h/day (10h00 to 13h00) (day 2 to 21) (Fig. 1a). For that purpose, pups were placed in warmed small plastic cages (stabilized to 32 °C). Weaning was set on day 21, and after that, all rats were fed *ad libitum* with chow (Nuvilab, CR-1 Quimtia SA, Paraná, Brazil). Body mass was recorded regularly throughout experimental period to control weight gain.

### Corticosterone radioimmunoassay

After anesthesia (Isoflurane, Cristalia, Itapira, SP, Brazil), blood was collected from abdominal aorta (Fig. 1a) in heparinized tubes (Liquemine, 5000 IU, Roche, Basel, Switzerland), centrifuged (15000 *g*, 10 min), and the plasma was kept at −20 °C. Euthanasia was conducted at 17h00 to prevent circadian variation. Total corticosterone was measured by RIA using Rat ^125^I corticosterone kit (ICN Biomedicals, Irvine, CA, USA). The amount of radioactivity was evaluated in a γ-scintillation counter (Wizard Automatic Gamma Counter, PerkinElmer, Waltham,, MA, USA). The intra-assay coefficient variation was 5.4% and the inter-assay coefficient variation was 9.3%.

### Stomach sample collection

Animals were euthanized for stomach collection (Fig. 1a). The organ was excised, opened along the lesser curvature, rinsed in 0.9% saline and submitted either to scraping mucosa (corpus region) or fixation in 10% formaldehyde. Samples that were isolated after scraping were used for extraction of RNA or proteins (total and nuclear contents). To that, the scraped mucosa was respectively collected in 100 µl of RNALater® (Life Technologies, Carlsbad, CA, USA), 10 nM phenylmethylsulfonyl fluoride (PMSF) (Merck, Darmstadt, Germany) in 20 mM Tris- buffered saline (TBS), and phosphate- buffered saline (PBS) added of protease inhibitors, to be immediately processed by Nuclear Extract Kit (Active Motif, Carlsbad, CA, USA).

### RNA isolation, cDNA synthesis and quantitative PCR

RNA was isolated with Trizol® reagent (Invitrogen, Carlsbad, CA, USA) combined to PureLink® RNA Mini Kit (Invitrogen), following manufacturer’s instruction. Total RNA concentration was determined using NanoDrop (Thermo Fisher Scientific, Waltham, MA, USA) and integrity was checked after eletrophoresis.

Single-stranded complementary DNAs (cDNAs) were synthesized from 3 µg of total RNA with Superscript III Reverse Transcriptase enzyme (200 U/μl, Invitrogen). Quantitative PCR (StepOne Plus Real Time PCR System, Applied Biosystems, Carlsberg, CA,USA) was used to detect the expression of genes that encode GAPDH, GR, Mist1, moesin and pepsinogen C. Amplification ran after qPCR with SYBR® (Life Technologies) and the specificity of primer sequences (listed in Supplementary Table S1) was confirmed via melt-curve analyses.

After cDNA dilution, 6 ng were used for qPCR with Power SYBR® Green PCR Master Mix (Thermo Fisher Scientific) to a total volume of 25 µl. Reactions were run using the following program: 10 min, 95 °C; 15 s, 95 °C, 1 min, 60 °C (40 cycles) followed by 95 °C, 15 s, 60 °C, 1 min, 95 °C, 15 s. Relative expression was calculated by 2^−∆∆Ct^ threshold method^63^ using values normalized to *Gapdh*.

### Protein extraction and western blot

Proteins were extracted after tissue lysis with NP- 40 buffer (20 mM Tris HCl pH 8.0, 135 mM NaCl, 1% NP-40 and 10% glycerol) containing protease and phosphatase inhibitors (1 mM PMSF, 0.45 mg/ml benzamidin, 1 mM leupeptin, 1 mM aprotinin and 5 mM sodium orthovanadate) (Sigma Aldrich, St. Louis, MO, USA). Concentration was determined^64^ for each animal, and thirty µg of proteins were separated into 12% SDS-PAGE, transferred onto nitrocellulose membranes (GE Healthcare, Wauwatosa, Wisconsin, USA), and blocked. The following antibodies (Santa Cruz Biotechnology, Santa Cruz, CA, USA) were used: rabbit anti- GR (5 µg/ml) (M20); goat anti- pepsinogen C (0.1 µg/ml) (I-19), monoclonal anti- Mist1 (5 μg/ml) (6E8), monoclonal anti- Moesin (5 μg/ml) (38/87) overnight at 4 °C. Monoclonal anti- β-actin (1 µg/ml) (Sigma Aldrich) was used as internal loading control. Reactions were developed with ECL Kit (GE Healthcare) and detected in X-ray films (Kodak, MXG-Plus, Rochester, NY, USA) (Supplementary Fig. 1). Densitometry was performed with ImageJ (1.37 v Software, NIH Public Domain) and bands were cropped to represent control and NMS groups. Gels were run in replicates to confirm results.

### GR binding assay

Nuclear proteins were obtained from gastric fresh samples scraped and placed in pre- chilled glass pestles to be manually homogenized according to instructions (Active Motif). Lysates were obtained (1X hypotonic buffer, added of proteases inhibitors), incubated on ice (15 min), centrifuged (850 *g*, 10 min, 4 °C), and the supernatant was transferred into pre- chilled micro- tube for efficiency checking. After a sequence of pellet washings in buffers for separation of cellular fractions, the supernatant containing the nuclear fraction was collected, separated in aliquots and stored at −80 °C. Nuclear and cytoplasmic protein concentration were determined^64^.

The protein quality and the cross contamination were checked after Western blot, using 10 or 5 µg of cytoplasmic and nuclear aliquots, respectively. Proteins were separated into 12% SDS- PAGE and blots were conducted as described to be incubated with rabbit anti- laminB1 (5 µg/ml) (Abcam, Cambridge, UK) and monoclonal anti- α- tubulin (5 µg/ml) (Sigma Aldrich) (overnight, 4 °C). Bands were detected with ECL kit, as described above.

Nuclear protein lysates were used for GR binding assay with TransAM GR Kit (Active Motif) that contains the reagents and a 96- well plate on which an oligonucleotide carrying a GR consensus binding site (5′-GGTACAnnnTGTTCT-3′) was immobilized. To detect GR binding, we added to each well: complete binding buffer, wild- type oligonucleotides (provided as a competitor for GR binding) or mutant oligonucleotides (provided as non- effector to GR binding) and nuclear extract (Fig. 3a). As positive controls, we ran HeLa nuclear extract, and to blank wells, we added complete binding buffer. Each sample was tested in duplicates. The plate was incubated, washed, and the wells were exposed to anti- GR (1:1000 1 h, RT), and then to a secondary antibody (same dilution). The developing solution was added for colorimetric reaction, which was stopped and read at 450 nm (EspectraMax, Molecular Devices, Sunnyvale, CA, USA).

### Immunohistochemistry

Non- serial sections were rehydrated and the peroxidase activity was blocked with 3% H_2_O_2_ in methanol. For GR, antigen retrieval was performed with 10 mM citric acid (pH 6.0 in microwave). Non- specific reaction was blocked with 10% normal goat or rabbit serum. Sections were incubated (4 °C, overnight, Santa Cruz Biotecnology) with: polyclonal rabbit anti- GR (10 μg/ml) (M20), polyclonal goat anti- pepsinogen C (1 μg/ml) (I-19). After adding secondary antibodies (Jackson ImmunoResearch Laboratories, West Grove, PA, USA), the peroxidase activity was developed with H_2_O_2_ and diaminobenzidine solution (Liquid DAB Kit, Dako, Carpinteria, CA, USA). Tissue sections were counterstained with Mayer’s hematoxylin. Negative controls were performed with normal serum to replace the primary antibodies.

In order to evaluate GR and pepsinogen C labeling indices, only sections that were longitudinally gastric mucosa in the corpus region were considered. Representative images were acquired using light microscope (BX51, Olympus, Canada) and Image ProPlus v.5.2 (Media Cybernetics, Silver Spring, MD, USA). The labeling index (LI) for GR was determined by counting 2,500 epithelial cells that were reactive or not to GR immunohistochemistry along the gastric gland. GR LI was estimated as labeled cells/total epithelial cells X 100.

In order to study pepsinogen C distribution in the gastric mucosa, we considered the whole bottom of glands at 18 days, as cells were not completely mature, and the zymogenic cells, as morphologically identified at gland base in 30-day-old rats. So, at 18 days, we determined the labeling index (LI) by counting 1,000 epithelial cells that were PgC- positive or not. At 30 days, we counted 1,000 zymogenic cells as labeled or not. PgC LI was estimated as labeled cells/total cells X 100 (35).

### Genomic analyses

The human genomic (hg19) regions for *Bhlha15* and *Pgc* were aligned with the rat (rn4) and mouse (mm10) genomes in the ECR browser (ecrbrowser.dcode.org). The evolutionarily conserved regions that appeared after alignment in Pgc coding and surrounding non-coding regions of all three species were submitted to the publicly available TRANSFAC professional V10.2 through rVista 2.0 and analyzed for the presence of the glucocorticoid response element (GRE-C), its half site matrix (GR_Q6-01) and high affinity binding site (GR_01) in the ECR of all three species (Supplementary Fig. S2).

The *Bhlha15* region is not annotated for the rat genome in the ECR browser, thus we first analyzed the evolutionarily conserved regions in the mouse and human sequences. The peaks that appeared in both species were submitted to TRANSFAC V10.2 through rVista 2.0, filtered for potential GR targets. Thereafter we used the mouse ECRs that presented potential GR targets to query the rat genome via BLAT in the USCS Genome browser, retrieving the homologous regions in the rat genome that corresponded to the mouse GR regulatory regions into the rat genome (rn4).

### Statistical analyses

The results were represented as means ± SD and analyzed by Student *t* test to evaluate differences between control and NMS groups. Statistical difference were considered when *P* < 0.05.

## Electronic supplementary material

Supplementary Information
