# Supplementary material for: Neonatal- maternal separation primes zymogenic cells in the rat gastric mucosa through glucocorticoid receptor activity
Source: Sci Rep. 2018 Jun 29;8:9823. doi: 10.1038/s41598-018-28223-1 (PMC6026145; doi:10.1038/s41598-018-28223-1)
Supplement: Supplementary file 1 — Supplementary Information [file 41598_2018_28223_MOESM1_ESM.docx]

Neonatal- maternal separation primes zymogenic cells in the rat gastric mucosa through glucocorticoid receptor activity.

Daniela Ogias, Isadora C. Rattes, Larissa Y. M. Hosoya, Juliana G. Zulian, Chao Yun Irene Yan, Patrícia Gama*

Department of Cell and Developmental Biology, Institute of Biomedical Sciences, University of Sao Paulo, São Paulo, Brazil.

Corresponding author: * P Gama- Department of Cell and Developmental Biology, Institute of Biomedical Sciences, University of Sao Paulo. Av Prof Lineu Prestes 1524, 05508-000, São Paulo, SP, Brazil. patgama@usp.br

**Supplementary Table S1.** Primers used for detection of genes amplified after qPCR with SYBR®.

| Encoded protein | | Gene | Forward | Reverse |
| --- | --- | --- | --- | --- |
| GAPDH | *Gapdh* | | AGTGCCAGCCTCGTCTCATAG | TAACCAGGCGTCCGATACG |
| GR | *Nr3c1* | | TACCCCCAACTAAATCGCTAATACA | TCGGTGCGGGTGAGATGT |
| Mist1 | *Bhlha15* | | GAACTTGTGCTTGGTCCATCCT | TCCCTATCCTGCGTTCACAAC |
| moesin | *moesin* | | TCAGAGCAGCCCGACAGTTT | AGTGGTGTTGGGCTGAATGG |
| pepsinogen C | *PgC* | | CTGGCTTCTTTGGCTATGACACT | CTCATTCTCACTCAGGCCAAACT |
| Mucin 6 | *Muc6* | | TACCTCTCACAGGAAGGACTACCAT | TCGTGTACTTGTTTTAGGTGGTGCTA |
| Sgk1 | *Sgk1* | | TATGGCCTGCCTCCGTTCT | GTGCCTTGCTGAGTTGTTGAT |
| H^+^/K^+^ ATPase | *Atp4a* | | TGCCCATCCGGTTCCA | TCCGGATCTCATCATAGACAAA |
|  |  | |  |  |

**Supplementary Figure S1.**

**
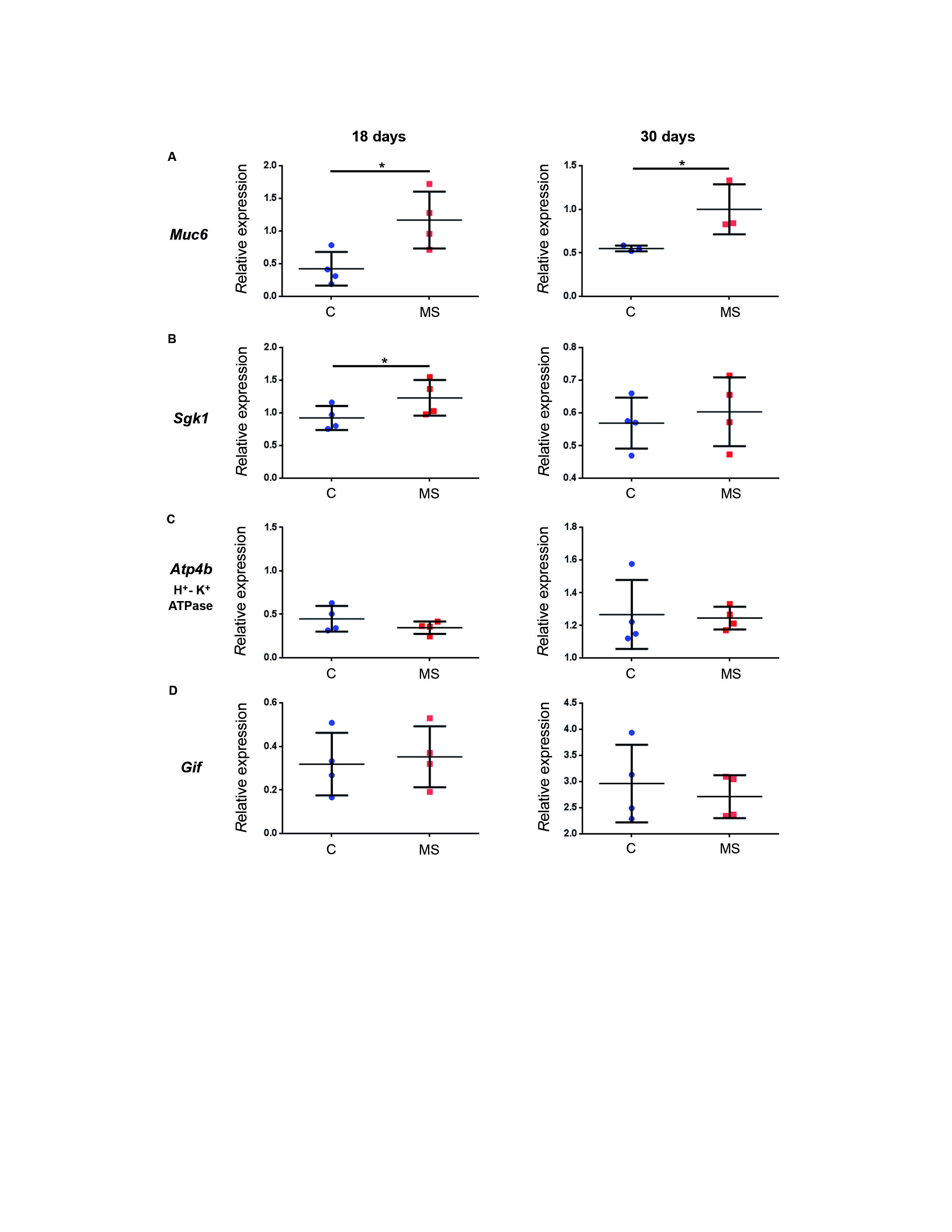
**

**Supplementary Figure S1.** (A) Gene expression was evaluated by RT- qPCR and shown as fold change of *Gapdh*. Primers are shown in Supplementary Table 1. *Muc6* (mucin 6), *Sgk1* (Sgk1), *Atp4b* (H^+^K^+^/ATPase proton pump), and *Gif* (gastric intrinsic factor). **P* < 0.05 for maternal separation (MS) as compared to control group by Student *t* test.

**Supplementary Figure S2.** Alignment of human, rat and mouse sequences in *Bhlha15* and *PgC* genes and identification of evolutionary conserved genomic regions that might function as GRE. The coding region is indicated by the bar on top of each figure: exons are in blue, untranslated regions in yellow and the intervening intron is a hashed thin blue line. Both the long and short splice forms of *PgC* are represented here. The evolutionarily conserved regions are identified by a pink bar and the degree of conservation is represented by the peaks under the bar. Each of the conserved regions shown here was analyzed for the presence of GREs in Transfac. The regions that contained GREs are highlighted by the blue boxes. Their corresponding genomic coordinates are listed on Supplementary Table SII.

**Supplementary Table S2**. Putative Glucocorticoid-responsive elements contained in evolutionary conserved genomic regions (ECR) in *Bhlha15* and *PgC* genes that might function as GRE. Regions in bold are also found in mice and regions in italic in humans.

| Gene | Region | ECR | Rat (rn4) genomic coordinates |
| --- | --- | --- | --- |
| bhlha15 | 1 | ***GR_01*** | ***Chr12: 10751318-10751348*** |
|  |  | ***GR_Q6_01*** | ***Chr12: 10751325-10751330*** |
|  | 2 | ***GR_Q6_01*** | **Chr12: 10751052-10751070** |
| PGC | 1 | GR_Q6_01 | Chr6: 8724192-8724200 |
|  | 2 | GR_Q6_01 | Chr6: 8725707-8725715 |
|  | 3 | GR_01 | Chr9:8728497-8728524 |
|  | 4 | ***GR_01*** | ***Chr9: 8729397-8729425*** |
|  |  | **GR_01** | **Chr9: 8729554-8729581** |
|  |  | **GRE_C** | **Chr9: 8729559-8729575** |
|  |  | **GR_Q6_01** | **Chr9: 8729560-8729568** |
|  |  | GRE_C | Chr9: 8729906-8729922 |
|  | 5 | GR_Q6_01 | Chr9:8732079-8732087 |

**GRE_C:** Glucocorticoid response element; **GR_Q6_01**: half site matrix; **GR_01**: high affinity binding site for glucocorticoid receptor.

**
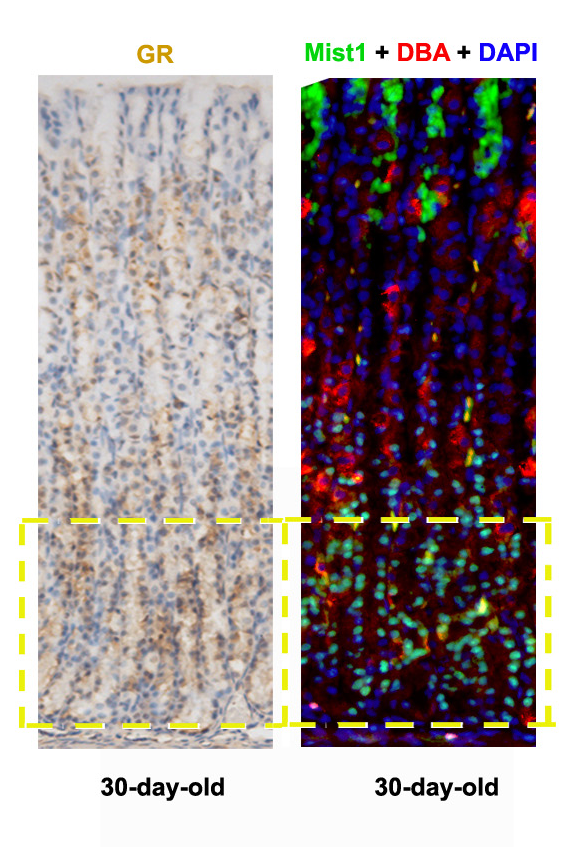
Supplementary Figure S3.**

**Supplementary Figure S3.** Localization of GR in the gastric mucosa at 30 days and the distribution of zymogenic and parietal cells. **Left:** Immunostaining and imaging for GR as described in Methods. Observe the distribution of cells along the gland, and their concentration at the neck- base interface. Development with DAB and counterstaining with Mayer´s hematoxylin. **Right:** ZC was identified by Mist1 immunofluorescence (mouse monoclonal antibody developed with FITC) and parietal cells were labeled by *Dolicus biflorus* agglutinin (DBA) lectin – TRITC. DAPI was used to counterstain the nuclei. Yellow squares denote the glandular transitional area (neck- base) and base, where most of GR+ cells concentrate. Images were obtained under immunofluorescence microscope (Zeiss). GR, Mist1 and DBA labeling were compared in different bright and dark fields to evaluate the localization of GR in the gland.

**
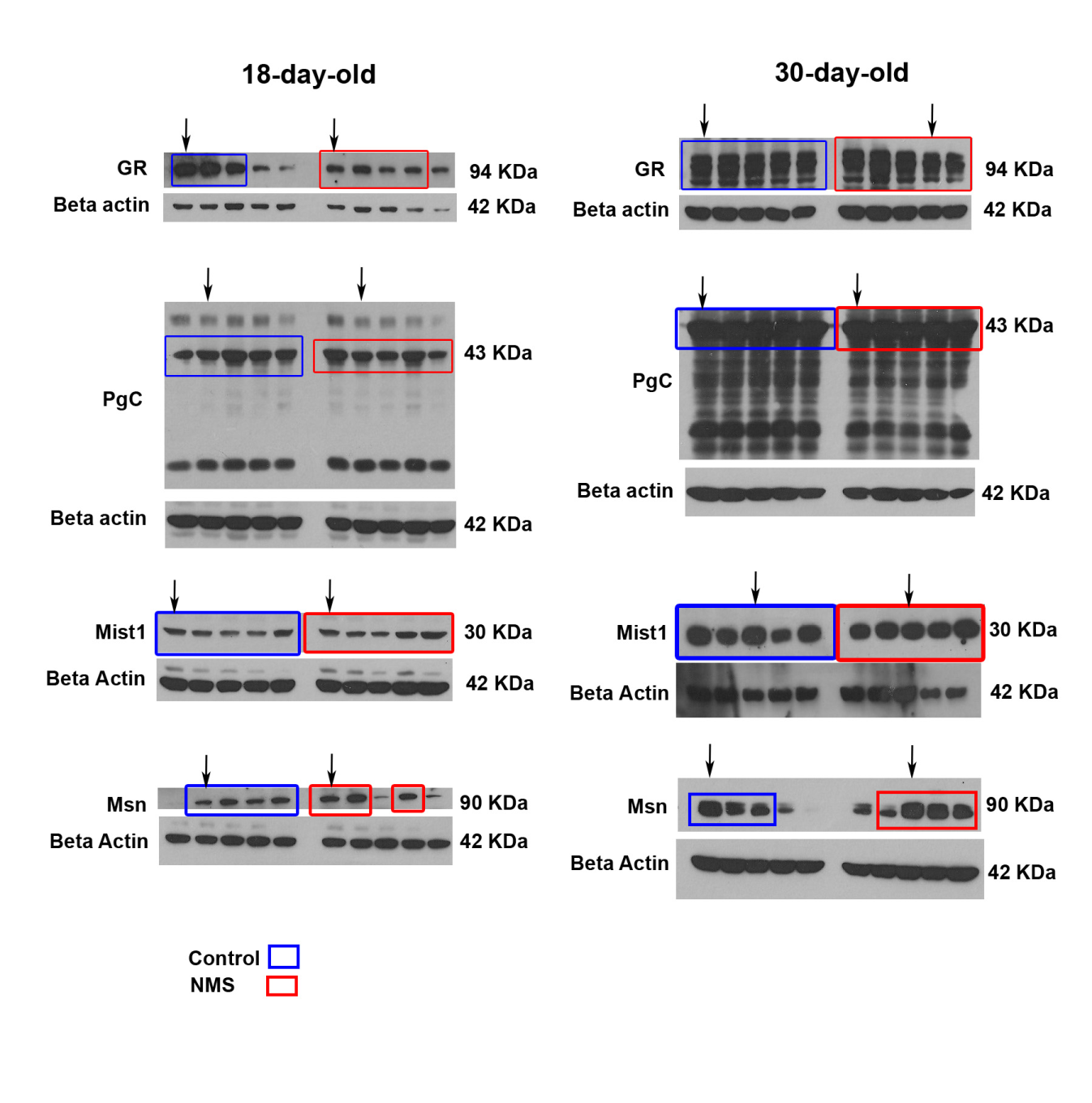
**

**Supplementary Figure S4**. Immunoblot representative films (blots were run in duplicates or triplicates for each protein and age) are shown in original sizes. Glucocorticoid receptor (GR in films 1 and 2), pepsinogen C (PgC in films 4 and 5), moesin (films 6 and 7), Beta- actin for each film as loading control. Films were developed with ECL kit, except for moesin that was developed with ECL Prime for higher resolution. C and NMS represent the control group and neonatal- maternal separation, respectively. Each band shows the result from one animal. Five samples were used for each treatment, and the samples used for densitometry (squares) in these films and those used as representative (arrows) are indicated.
